# Reclassification of Hepatocellular Cancer With Neural-Related Genes

**DOI:** 10.3389/fonc.2022.877657

**Published:** 2022-05-13

**Authors:** Yi-Gan Zhang, Ming-Zhu Jin, Xiao-Ran Zhu, Wei-Lin Jin

**Affiliations:** ^1^The First Clinical Medical College of Lanzhou University, Lanzhou, China; ^2^Institute of Cancer Neuroscience, Medical Frontier Innovation Research Center, The First Hospital of Lanzhou University, The First Clinical Medical College of Lanzhou University, Lanzhou, China; ^3^Department of Gynecology and Obstetrics, Xinhua Hospital Affiliated to Shanghai Jiao Tong University School of Medicine, Shanghai, China

**Keywords:** neural-related genes (NRGs), liver cancer, nerve-cancer crosstalk, immune infiltration, biomarker

## Abstract

Neural infiltration is a critical component of the tumor microenvironment; however, owing to technological limitations, its role in hepatocellular cancer remains obscure. Herein, we obtained the RNA-sequencing data of liver hepatocellular carcinoma (LIHC) from The Cancer Genome Atlas database and performed a series of bioinformatic analyses, including prognosis analysis, pathway enrichment, and immune analysis, using the R software packages, Consensus Cluster Plus and Limma. LIHC could be divided into two subtypes according to the expression of neural-related genes (NRGs); moreover, there are statistic differences in the prognosis, stage, and immune regulation between the two subtypes. The prognostic model showed that high expression of NRGs correlated with a poor survival prognosis (*P*<0.05). Further, *CHRNE*, *GFRA2*, *GFRA3*, and *GRIN2D* was significantly correlated with LIHC clinical prognosis, clinical stage, immune infiltration, immune response, and vital signaling pathways. There was nerve-cancer crosstalk in LIHC. A reclassification of LIHC based on NRG expression may prove beneficial to clinical practice. *CHRNE*, *GFRA2*, *GFRA3*, and *GRIN2D* may serve as potential biomarker for liver cancer prognosis or immune response.

## Introduction

According to the annually published statistics of the American Cancer Society, liver cancer has been among the ten leading cancer types with respect to incidence and mortality rates (1). Despite the majority of the risk factors, such as hepatitis virus infection, and excess alcohol consumption, being modifiable, the incidence of liver cancer in women is estimated to rise (1).

The liver is under neural control through sympathetic and parasympathetic nerves. For example, the sympathetic nerve fiber modulator neuregulin 4 (NRG4) negatively regulates brown adipocyte differentiation, hepatic steatosis, and hepatic lipogenesis in a cell-autonomous manner (2). Recently, Mizuno et al. have shown that the nerve fiber areal ratios (NFARs) for total nerve fibers and sympathetic nerve fibers were reduced in the liver biopsy samples from patients with viral hepatitis and liver fibrosis, and NFAR recovery might be seen after the antiviral treatment for hepatitis C as well as the improvement of liver fibrosis (3). In general, previous studies have shown that the hepatic nervous system has several modulatory effects on liver glucose metabolism, lipid metabolism, bile secretion, repair, and regeneration (4).

Mounting evidence suggests a nerve–cancer crosstalk in liver cancer. Yong et al. uncovered that the mesencephalic astrocyte-derived neurotrophic factor inhibited epithelial–mesenchymal transition and liver cancer progression *via* the suppression of the nuclear factor kappa-light-chain-enhancer of activated B cells/Snail signaling, cultivating a nexus among endoplasmic reticulum stress, and liver cancer inflammation and progression (5). Additionally, Lin et al. demonstrated that the nerve growth factor influenced cancer invasion and metastasis by regulating the polarity and motility of liver cancer cells (6). Thus far, the research on nerve–cancer crosstalk is at an early stage, and the functional role of neural-related genes (NRGs) in liver cancer are obscure. We proposed that neuroregulation is associated with a series of biological processes in the liver, including cancer progression, metabolism, and immunoregulation. In this study, we attempted to provide a comprehensive analysis of the involvement of NRGs in the clinical prognosis as well as disease subgroups and interpret their biological and clinical significance in critical signaling pathways.

## Materials and Methods

### Identification of Neural-Related Genes and Subtype Classification

We obtained the original data and corresponding clinical information of 371 liver hepatocellular carcinoma (LIHC) cases from The Cancer Genome Atlas (TCGA) database (https://portal.gdc.com). A total of 42 NRGs were identified from a previous comprehensive review (7). Four NRGs, *ADRB3*, *CHRM1*, *CHRM2*, and *CHRNA9*, were eliminated since they were not expressed in LIHC. The R software package ConsensusClusterPlus (v1.54.0) (8) was applied for consensus analysis. The PAC structure of the tool identifies the default cluster number and is repeated 100 times to extract 80% of the sample, clusterAlg = “hc”, and innerLinkage= ‘ward. D2’. Heat map clustering was analyzed using the R software package pheatmap (v1.0.12). The genes with a variance above 0.1 were retained in the gene expression heat map. If the number of input target genes was more than 1,000, the top 25% genes were extracted and displayed, after sequencing, from a large to small variance.

### Comparison of Clinical Characteristics

Two subtypes were gained following a consensus analysis, and clinical information was downloaded. For *CHRNE*, *GFRA2*, *GRIN2D*, cutoff-high (top 25%) and cutoff-low (bottom 25%) and for *GFRA3*, cutoff-high (25%) and cutoff-low (25%) were used as thresholds. R software package (v4.0.3) ggplot2 (9) and pheatmap were applied. Significance p-values were analyzed by the chi-square test, where the size of the value was taken -log10 (p-value), and, if marked with *, it represents a significant difference in the distribution of this clinical characteristic in the corresponding two groups (p < 0.05).

### Differential Expression Analysis

The R software package Limma (v3.40.2) was used to study the differential expression of mRNA (10). Adjusted *p*-values were analyzed in TCGA or GTEx to correct for false- positive results. Adjusted *P*<0.05 with log2(FC) (multiple change)>1 or log2(FC) (multiple change) <-1 was defined as the threshold for the differential expression of mRNA. The differential expression analysis between subtypes was commonly used: C1 *vs.* C2. *CHRNE*, *GFRA2*, and *GRIN2D* used cutoff-high (top 25%) and cutoff-low (bottom 25%) as expression thresholds. GFRA3 used cutoff-high (25%) and cutoff-low (25%) as thresholds, since they had significantly low expression in LIHC.

### Enrichment Analysis

The R software package ClusterProfiler (11) was used to carry out Gene Ontology (GO) (12), and the Kyoto Encyclopedia of Genes and Genomes (KEGG) (13) was used for the analysis of potential mRNA.

### Establishment of a Prognostic Model Based on Neural-Related Genes

Based on the clinical information from 371 LIHC cases from the TCGA database, we generated Kaplan–Meier (KM) plots with log-rank *P-*value and time-dependent receiver operating characteristic (ROC) analysis to compare the predictive accuracy and risk score of 38 neural-related genes. The least absolute shrinkage and selection operator (LASSO) regression algorithm was used for feature selection, and 10-fold cross validation was used, lambda. min=0.071 (14). The R software package glmnet was used for the above analysis. For KM curves, *p*-values and hazard ratios with a 95% confidence interval (CI) were determined using a log rank test and univariate Cox proportional hazard regression. All the above analyses were performed using the R software package (v4.0.3). *P*<0.05 was considered as a statistical difference.

### Stemness Analysis

The one-class logistic regression (OCLR) algorithm, invented by Malta et al., was used to evaluate the stemness of mRNA (15).

### Immune Infiltration Analysis

CIBERSORT and EPIC from the R software package immunedeconv (https://grst.github.io/immunedeconv) were applied to analyze the immune infiltration of different subtypes (16). The R software packages (v4.0.3) ggplot2 and pheatmap were used for visualization.

### Immune Checkpoint Genes Analysis and Response Prediction

We tested the correlation of specific neural-related gene expression and 8 commonly used immune checkpoint genes (ICGs) (*e.g.*, *CD274*, *CTLA4*, *HAVCR2*, *LAG3*, *PDCD1*, *PDCD1LC2*, *SIGLEC15* and *TIGIT*). The R software packages (v4.0.3) ggplot2 and pheatmap were used for visualization. The TIDE algorithm was applied to predict the therapeutic response to immune checkpoint blockades (ICBs) (16).

## Results

### Identification of Liver Hepatocellular Carcinoma Subtypes Based on Neural-Related Genes

**Figure 1:**
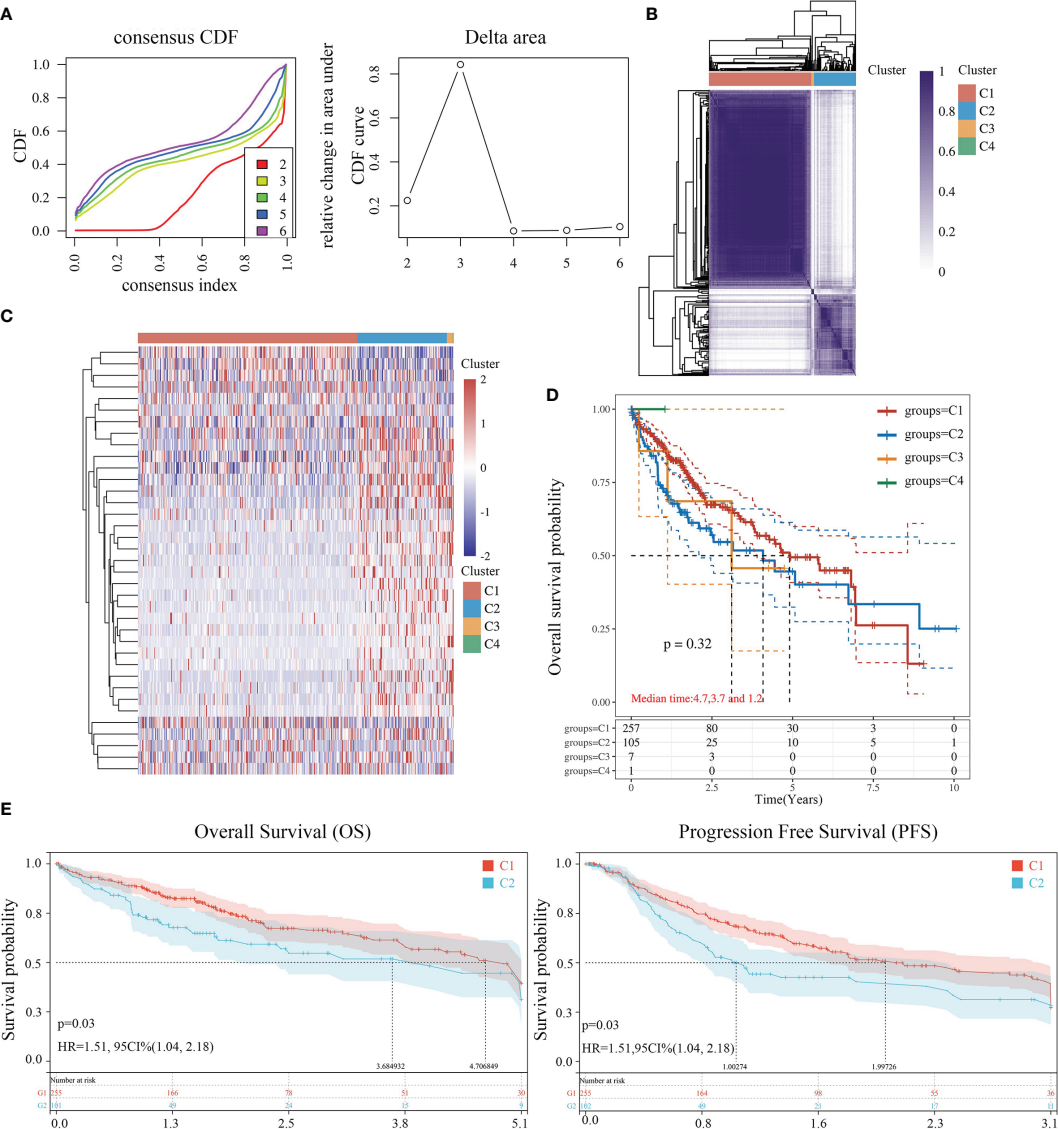
Identification of LIHC subtypes based on NRGs using ConsensusClusterPlus. **(A)** CDF curve and delta area curve of consensus clustering. **(B)** Heat map of consensus clustering solution. Rows and columns represent samples, and the colors represent different categories. **(C)** Heat map of NRG expression in different subtypes: red corresponds to high expression, while blue corresponds to low expression. **(D)** The KM survival curve of different samples in TCGA data sets. **(E)** The OS (5 years) and PFS KM curves (3 years) of groups C1 and C2.

**Figure 2:**
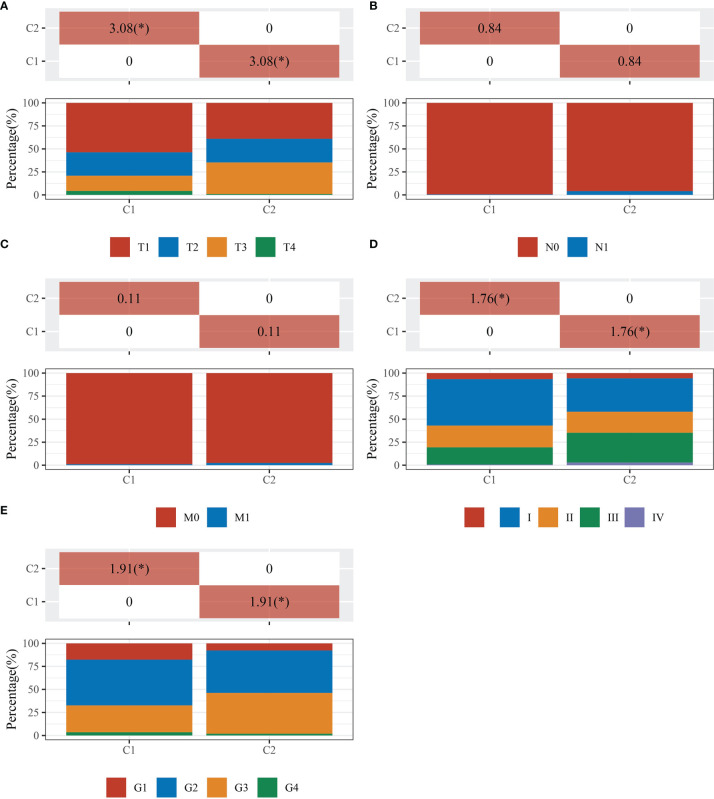
Comparisons of clinical characteristics between C1 and C2. The distribution of clinical characteristics of group C1 and C2. The horizontal axis represents different groups of samples. The vertical axis represents the percentage of clinical information contained in the corresponding grouped samples. The table above shows the *p*-value (-log10) of clinical feature significance in the two groups, which calculated by the chi-square test. The * mark indicates a significant difference in clinical features between the two groups (*P*<0.05). **(A)** T category. **(B)** N category. **(C)** M category. **(D)** TNM staging. **(E)** Pathological grading.

The data of 38 NRGs in LIHC were collected from the TCGA database. As mentioned previously, *ADRB3*, *CHRM1*, *CHRM2*, and *CHRNA9* were eliminated since they were not expressed in LIHC. For identification of subtypes, the R software package ConsensusCluster Plus (V1.5.4.0) was applied to obtain 4 subtypes: group 1 (C1), group 2 (C2), group 3 (C3), and group 4 (C4) (**Figures 1A–C**). The groups C3 and C4 were excluded from this study due to limited sample size, which might lead to a confined biological or clinical value. Ten years post-follow-up, the overall survival (OS) and progression-free survival (PFS) of the four groups had no statistic difference (*P*>0.05) (**Figure 1D**). However, the 5-year OS and 3-year PFS of C1 were better than C2 (*P*=0.03; *P*=0.03) (**Figure 1E**). They could be attributable to the withdrawal of C1 during the sixth-year follow-up. In addition, C1 had a better T category, clinical staging, and pathological grading with a significant statistical difference (*P*<0.05) (**Figure 2** and **Table 1**).

**Table 1:** Comparisons of clinical characteristics between C1 and C2.

C1 vs C2				
	Characteristics	C1	C2	P_value
Status	Alive	173	63	
	Dead	85	42	0.248
Age	Mean (SD)	61.4 (12.1)	54.8 (15.4)	
	Median [MIN, MAX]	63 [16,90]	56 [17,85]	0
Gender	FEMALE	70	45	
	MALE	188	60	0.005
Race	AMERICAN INDIAN	1	1	
	ASIAN	104	50	
	BLACK	14	3	
	WHITE	129	51	0.512
pT_stage	T1	137	41	
	T2	65	25	
	T3	24	20	
	T3a	14	14	
	T3b	4	2	
	T4	11	1	
	TX	1		
	T2a		1	
	T2b		1	0.004
pN_stage	N0	175	71	
	N1	1	3	
	NX	82	30	0.111
pM_stage	M0	181	81	
	M1	2	2	
	MX	75	22	0.201
pTNM_stage	I	130	38	
	II	61	24	
	III	2	1	
	IIIA	34	29	
	IIIB	6	2	
	IIIC	6	2	
	IV	1	1	
	IVB	1	1	
	IVA		1	0.066
Grade	G1	45	8	
	G2	127	48	
	G3	74	46	
	G4	9	2	0.012
new_tumor_event_type	Primary	5	5	
	Recurrence	113	48	0.324
Radiation_therapy	Non-radiation	178	58	
	Radiation	2	2	0.56
History_of_neoadjuvant_treatment	Neoadjuvant	1	1	
	No neoadjuvant	257	104	1
Therapy_type	Ancillary	1		
	Chemotherapy	21	8	
	Chemotherapy : Targeted Molecular therapy	1	1	
	Targeted Molecular therapy	3	2	
	Chemotherapy : Hormone Therapy		1	
	Other. specify in notes		1	0.709

### Differential Expression Analysis and Enrichment of C1 and C2

**Figure 3:**
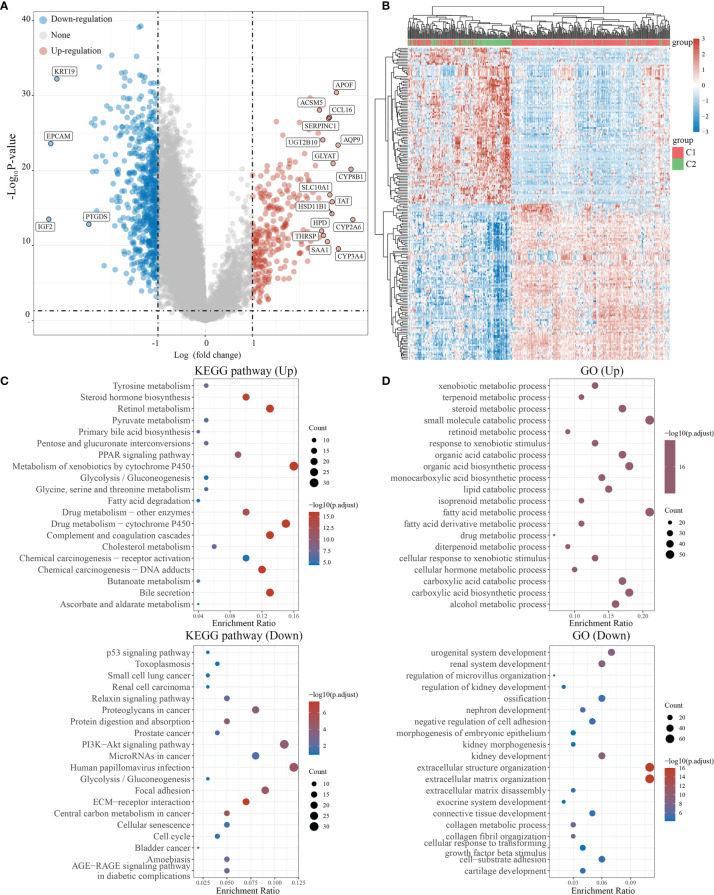
Differential expression and enrichment analysis of C1 and C2. **(A)** The volcano plot shows the differential gene expression of C1 and C2 drawn with fold-change values and adjusted P. **(B)** Heat map showing differential gene expression (only 50 genes were displayed because of the large quantity of the genes). **(C, D)** KEGG and GO analysis showed the upregulated/downregulated pathways of the C1 compared with C2. *P*<0.05 or FDR<0.05 is considered to be meaningful.

We used the R software package Limma (v3.40.2) to analyze differentially expressed genes in C1 and C2. Those with FC>2 and *P*<0.05 were interpreted as differentially expressed genes (DEGs). Compared to C2, C1 had 622 downregulated and 262 upregulated DEGs. Among 38 NRGs, C1 possessed 27 downregulated (*e.g.*, *CHIN3A*), 5 upregulated (*e.g., CHRNA4*), and 6 unregulated (*e.g., CHRM3*) DEGs compared to C2 (**Figures 3A, B**, **S1**). Meanwhile, KEGG and GO analyses were carried out to further assess signaling pathways in C1 and C2 (**Figures 3C, D**). KEGG analysis revealed that compared to C2, C1 showed the activation of signaling pathways for liver metabolism, including bile secretion, cholesterol metabolism, glycolysis/gluconeogenesis, and chemical carcinogenesis (DNA adducts and receptor activation), while a suppression of cancer-associated signaling pathways such as bladder cancer, the cell cycle, central carbon metabolism in cancer, and PI3K-Akt signaling pathway (**Figure 3C**). GO analysis suggested that compared to C2, C1 had activated the liver metabolic process (*e.g*., alcohol metabolic process, fatty acid metabolic process) and a suppression of growth factor beta stimulus, negative regulation of cell adhesion, extracellular matrix organization, which were regarded as critical processes in promoting cancer growth and metastasis (**Figure 3D**).

### Analysis of the Immune Status of C1 and C2

**Figure 4:**
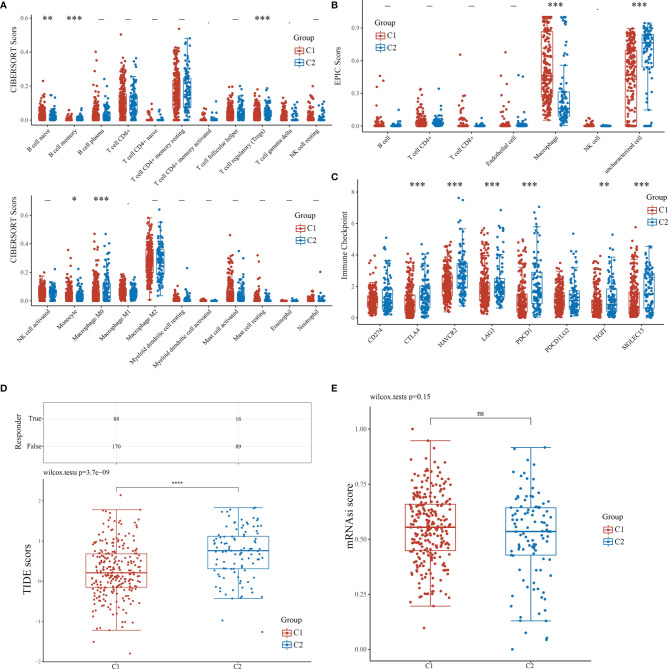
Comparisons of immune status and stemness between C1 and C2. **(A, B)** Comparison of C1 and C2 in immune infiltration obtained using CIBERSORT and EPIC algorithm. The horizontal axis represents different immune cells; the vertical axis represents the immune scores (*P<0.05, **P<0.01, ***P<0.001). **(C)** Comparison of immune-checkpoint gene expression in C1 and C2. The horizontal axis shows different immune checkpoint genes; the vertical axis shows the expression level (*P<0.05, **P<0.01, ***P<0.001). **(D)** Statistical table showing immune response and the distribution of immune response scores of the different groups with the predicted outcome. **(E)** Comparison of C1 and C2 in stemness demonstrated with mRNAsi score using the OCLR algorithm.

For the evaluation of the immune infiltration of C1 and C2, we used immunedeconv, an R software package based on the integration of CIBERSORT, EPIC, MCP-counter, quanTIseq, TIMER, and xCell (16). During the analysis, we used CIBERSORT and EPIC, which were the most used algorithms. CIBERSORT analysis revealed significant differences in the naïve B cell (*P*<0.01), memory B cell (*P*<0.001), regulatory T cell (Tregs)(*P*<0.001), monocyte (*P*<0.05), and macrophage M0 (*P*<0.001), suggesting that C1 exhibited stronger immunosuppression when compared with C2 (**Figure 4A**). EPIC further confirmed that C1 and C2 had significant difference in macrophage infiltration (*P*<0.001); however, there were insufficient data on macrophage subtypes (**Figure 4B**). We also applied R software packages ggplot2 and pheatmap to analyze ICGs in the two subtypes and showed that *CTLA4*, *HAVCR2*, *LAG3*, *PDCD1*, *SIGLEC15* (*P*<0.001), and *TIGIT* (*P*<0.01) were downregulated in C1 when compared to C2 (**Figure 4C**). The TIDE algorithm, used to predict cancer immune response, was also used herein (16). The TIDE score was higher in C2 than C1, with significant difference (*P*<0.001), indicating that C1 might achieve more clinical benefit upon ICBs (**Figure 4D**). The OCLR algorithm showed that the stemness of C1 and C2 had no statistical difference (*P*>0.05) (16) (**Figure 4E**).

### Prognostic Analysis of Neural-Related Gene Expression in Liver Hepatocellular Carcinoma

**Figure 5:**
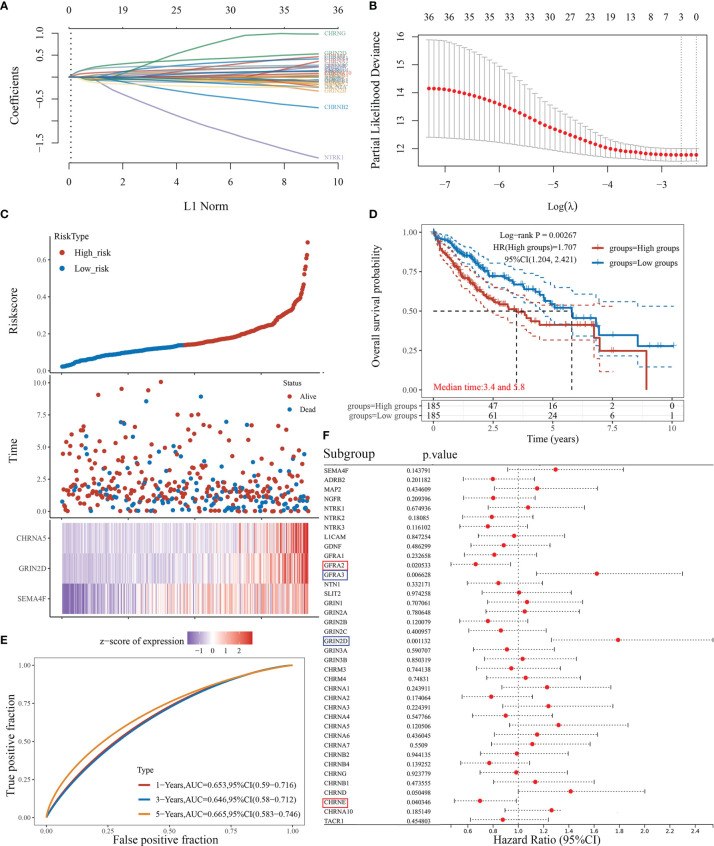
The prognostic model of liver cancer based on 38 neural-related genes. **(A)** The coefficients of 38 neural-related genes shown by lambda parameter. The vertical axis represents the coefficients of independent variables, and the horizontal axis represents lambda. **(B)** LASSO COX regression model was used to draw the partial likelihood deviance versus log(λ). **(C)** The relationship between risk score and living status. The dotted line represents the risk score and divides the patients into high-risk and low-risk groups. A scatter diagram is in the middle, and the heat map of gene expression is down below. **(D)** KM survival curve of the risk model in TCGA data set. Different groups were tested by log rank and HR (high exp), and represent the risk factors of the high expression group compared with the low expression group. **(E)** The ROC curve of the risk model and AUC at various timepoints (1 year, 3 years, 5 years). **(F)** The univariate COX analysis, the *p*-value of clinical features, and hazard ratio (HR) confidence interval of 38 neural-related gene expressions.

We attempted to elucidate the relationship between NRGs and LIHC prognosis using the LASSO regression algorithm, an R software package conducive to dimension reduction analysis and prognostic gene model construction (14). The results showed that the increased expression of neural-related genes was positively associated with the poor prognosis of LIHC (*P*<0.05) (**Figures 5A–D**). The expressions of 38 NRGs were potentially prognostic biomarkers for LIHC patients: the area under the curve (AUC) was 0.653 for 1-year, 0.646 for 3-year, and 0.665 for 5-year ROC curves (**Figure 5E**). Individual prognosis analysis showed that 4 of the 38 NRGs had a correlation with LIHC prognosis (*P*<0.05) (**Figure 5F**). The genes *CHRNE* and *GFRA2*, and *GFRA3* and *GRIN2D* correlated positively and negatively with LIHC prognosis, respectively (**Figure 5F**). When the four aforementioned NRGs were included in the prognostic model, the AUC value was 0.672 for 1-year, 0.618 for 3-year, and 0.679 for 5-year ROC curves (**Figure S2E**). These results demonstrated that the expressions of specific neural-related genes including *CHRNE*, *GFRA2*, *GFRA3*, and *GRIN2D* might be potential biomarkers for LIHC prognosis.

### Correlation Between CHRNE/GFRA2/GFRA3/GRIN2D Expression and Clinical Features

We classified the expression of *CHRNE*, *GFRA2*, *GFRA3*, and *GRIN2D* into high and low expression groups based on the RNA-sequencing (RNA-seq) and clinical data collected from the TCGA database. A cutoff-high (top 25%) for high expression, while cutoff-low (bottom 25%) for low expression for *CHRNE*, *GFRA2* and *GRIN2D* was defined. Moreover, a cutoff high (50%) and cutoff low (50%) was used as the expression threshold for *GFRA3* due to its relatively low expression. The results showed that high expression of *CHRNE* was positively correlated with the T category and TNM staging (**Figure S3A** and **Table 2**); high expression of *GFRA2* was positively correlated with the T category (**Figure S3B** and **Table 3**); high expression of *GFRA3* was negatively correlated with the T category and pathological grading (**Figure S3C** and **Table 4**); high expression of *GRIN2D* was negatively correlated with the T category, TNM staging, and pathological grading (**Figure S3D** and **Table 5**).

**Table 2:** Comparisons of clinical characteristics between high and low *CHRNE* expression groups.

CHRNE-HIGH vs CHRNE-LOW				
	Characteristics	CHRNE-HIGH	CHRNE-LOW	P_value
Status	Alive	61	52	
	Dead	32	41	0.23
Age	Mean (SD)	57 (14)	60.2 (13.5)	
	Median [MIN, MAX]	58 [17,82]	64 [23,85]	0.115
Gender	FEMALE	37	25	
	MALE	56	68	0.087
Race	ASIAN	40	45	
	BLACK	3	6	
	WHITE	48	37	
	AMERICAN INDIAN		2	0.263
pT_stage	T1	56	36	
	T2	15	26	
	T3	9	15	
	T3a	8	6	
	T3b	2	4	
	T4	3	5	
	T2a		1	0.069
pN_stage	N0	66	67	
	N1	2	1	
	NX	25	24	0.837
pM_stage	M0	66	75	
	M1	1	1	
	MX	26	17	0.293
pTNM_stage	I	52	34	
	II	14	23	
	IIIA	13	21	
	IIIB	4	3	
	IIIC	2	5	
	IVB	1		
	IV		1	0.055
Grade	G1	18	13	
	G2	44	38	
	G3	29	36	
	G4	2	4	0.45
new_tumor_event_type	Primary	3	2	
	Recurrence	40	43	0.958
Radiation_therapy	Non-radiation	65	55	
	Radiation	1		
History_of_neoadjuvant_treatment	Neoadjuvant	1	1	
	No neoadjuvant	92	92	1
Therapy_type	Chemotherapy	8	8	
	Other. specify in notes	1		
	Chemotherapy : Hormone Therapy : Other. specify in notes		1	
	Targeted Molecular therapy		2	

**Table 3:** Comparisons of clinical characteristics between high and low *GFRA2* expression groups.

GFRA2-HIGH vs GFRA2-LOW				
	Characteristics	GFRA2-HIGH	GFRA2-LOW	P_value
Status	Alive	59	54	
	Dead	34	39	0.548
Age	Mean (SD)	59.4 (14.5)	60.7 (12.8)	
	Median [MIN, MAX]	61 [17,90]	62 [24,84]	0.501
Gender	FEMALE	31	39	
	MALE	62	54	0.289
Race	ASIAN	37	37	
	BLACK	5	2	
	WHITE	47	49	
	AMERICAN INDIAN		2	0.516
pT_stage	T1	51	29	
	T2	20	29	
	T3	9	14	
	T3a	7	11	
	T4	3	5	
	TX	1		
	T2a		1	
	T3b		4	0.038
pN_stage	N0	65	62	
	NX	28	31	0.753
pM_stage	M0	66	67	
	MX	27	24	
	M1		2	0.812
pTNM_stage	I	48	28	
	II	20	25	
	III	1	1	
	IIIA	14	22	
	IIIC	1	3	
	IIIB		5	
	IV		2	0.076
Grade	G1	16	12	
	G2	42	46	
	G3	28	31	
	G4	4	3	0.795
new_tumor_event_type	Primary	5	1	
	Recurrence	38	46	0.167
Radiation_therapy	Non-radiation	57	60	
	Radiation	2	1	0.977
History_of_neoadjuvant_treatment	No neoadjuvant	93	92	
	Neoadjuvant		1	
Therapy_type	Chemotherapy	10	11	
	Ancillary		1	
	Chemotherapy : Hormone Therapy : Other. specify in notes		1	
	Targeted Molecular therapy		2	

**Table 4:** Comparisons of clinical characteristics between high and low *GFRA3* expression groups.

GFRA3-HIGH vs GFRA3-LOW				
	Characteristics	GFRA3-HIGH	GFRA3-LOW	P_value
Status	Alive	112	129	
	Dead	74	56	0.07
Age	Mean (SD)	55.9 (13.9)	63 (12.2)	
	Median [MIN, MAX]	57 [17,85]	65 [16,90]	0
Gender	FEMALE	66	55	
	MALE	120	130	0.284
Race	AMERICAN INDIAN	1	1	
	ASIAN	95	63	
	BLACK	10	7	
	WHITE	77	107	0.008
pT_stage	T1	77	104	
	T2	49	43	
	T2a	1		
	T2b	1		
	T3	29	16	
	T3a	18	11	
	T3b	3	3	
	T4	8	5	
	TX		1	0.061
pN_stage	N0	136	116	
	N1	3	1	
	NX	46	68	0.033
pM_stage	M0	141	125	
	M1	2	2	
	MX	43	58	0.203
pTNM_stage	I	74	97	
	II	47	39	
	III	1	2	
	IIIA	42	23	
	IIIB	3	5	
	IIIC	6	3	
	IV	2		
	IVA	1		
	IVB		2	0.048
Grade	G1	22	33	
	G2	82	95	
	G3	73	49	
	G4	9	3	0.013
new_tumor_event_type	Primary	8	2	
	Recurrence	90	73	0.228
Radiation_therapy	Non-radiation	113	127	
	Radiation	1	3	0.709
History_of_neoadjuvant_treatment	Neoadjuvant	2		
	No neoadjuvant	184	185	
Therapy_type	Ancillary	1		
	Chemotherapy	15	14	
	Chemotherapy : Hormone Therapy	1		
	Chemotherapy : Targeted Molecular therapy	2		
	Other. specify in notes	1		
	Targeted Molecular therapy	3	2	
	Chemotherapy : Hormone Therapy : Other. specify in notes		1	1

**Table 5:** Comparisons of clinical characteristics between high and low *GRIN2D* expression groups.

GRIN2D-HIGH vs GRIN2D-LOW				
	Characteristics	GRIN2D-HIGH	GRIN2D-LOW	P_value
Status	Alive	56	72	
	Dead	37	21	0.018
Age	Mean (SD)	56.4 (13)	61.9 (12.7)	
	Median [MIN, MAX]	57 [17,85]	65 [24,85]	0.004
Gender	FEMALE	35	24	
	MALE	58	69	0.115
Race	ASIAN	50	38	
	BLACK	3	4	
	WHITE	39	49	
	AMERICAN INDIAN		1	0.233
pT_stage	T1	34	53	
	T2	25	23	
	T2a	1		
	T2b	1		
	T3	18	8	
	T3a	9	6	
	T3b	1	3	
	T4	4		0.05
pN_stage	N0	66	66	
	N1	3		
	NX	23	27	0.752
pM_stage	M0	74	66	
	M1	1	1	
	MX	18	26	0.384
pTNM_stage	I	32	50	
	II	24	22	
	IIIA	23	14	
	IIIB	4	2	
	IIIC	4		
	IV	1	1	
	IVA	1		
	III		1	0.149
Grade	G1	6	20	
	G2	44	45	
	G3	37	27	
	G4	5	1	0.008
new_tumor_event_type	Primary	5	2	
	Recurrence	38	43	0.395
Radiation_therapy	Non-radiation	51	69	
	Radiation	1	2	1
History_of_neoadjuvant_treatment	No neoadjuvant	93	93	
Therapy_type	Chemotherapy	4	7	
	Chemotherapy : Hormone Therapy : Other. specify in notes	1		
	Chemotherapy : Targeted Molecular therapy	1	1	
	Chemotherapy : Hormone Therapy		1	
	Targeted Molecular therapy		1	1

### Biological Significance of CHRNE/GFRA2/GFRA3/GRIN2D in Liver Hepatocellular Carcinoma

The LIHC data was further classified according to the expression levels of the NRGs: *CHRNE*, *GFRA2*, *GFRA3* and *GRIN2D*. Furthermore, GO and KEGG analyses were performed for each group. The criterion for low and high expression remains the same as mentioned above.

**Figure 6:**
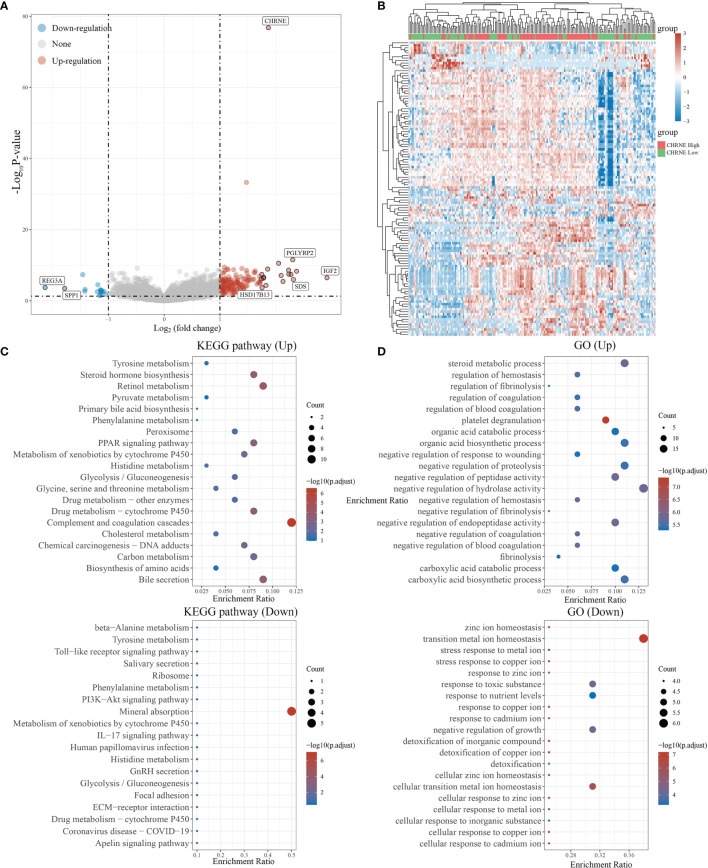
Differential expression and enrichment analysis of high and low *CHRNE* expression groups. **(A)** The volcano plot shows the differential gene expression of CHRNE high expression group and CHRNE low expression group was drawn with fold-change values and adjusted P. **(B)** Differential gene expression showed by heatmap (only 50 genes were displayed because of the large quantity of the genes); **(C, D)** KEGG and GO analysis showed the upregulated/downregulated pathways of the CHRNE high expression group compared with the low expression group. When P <0.05 or FDR <0.05 is considered to be enriched to a meaningful pathway.

In LIHC with high *CHRNE* expression, 150 genes were unregulated and 16 genes were downregulated (FC>2, *P*<0.05) (**Figures 6A, B**), while a low expression of *CHRNE* had an upregulation of genes associated with physiological functions of the liver (*e.g.*, bile secretion, cholesterol metabolism, drug metabolism) and activation processes, such as the regulation of coagulation; downregulation of tumor-promoting pathways, including the PI3K-Akt signaling pathway; and suppression of processes like transition metal ion homeostasis (**Figures 6C, D**).

In LIHC with a high *GFRA2* expression, 195 genes were upregulated and 19 genes were downregulated (FC>2, *P*<0.05) (**Figures S4A, B**), while a low expression of *GFRA2* in LIHC-activated cytokine and cytokine receptors, Th1 and Th2 cell differentiation, and Th17 cell differentiation signaling pathways, which are closely related to immune regulation, were observed. Pro-tumor pathways, such as the HIF-1 signaling pathway and p53 signaling pathway, were suppressed in the group with high *GFRA2* expression. GO analysis exhibited similar results: activated immune regulation-associated processes like T-cell activation, leukocyte proliferation, regulation of T-cell activation, and inhibited cell maturation (**Figures S4C, D**).

In LIHC with a high *GFRA3* expression, 144 genes were upregulated and 56 genes were downregulated (FC>2, *P*<0.05) (**Figures S5A, B**) For the LIHC group with a high *GFRA3* expression, widely recognized critical oncogenes in liver cancer, such as *AFP*, *IGF2*, and liver cancer-associated pathways (*e.g*., cell cycle, forkhead box O, signaling pathway, hepatitis B, MAPK signaling pathway, VEGF signaling pathway, and p53 signaling pathway) were unregulated, while the cell proliferation-related processes (e.g., chromosome segregation, mitotic nuclear division, spindle organization) were activated. However, pathways like bile secretion and processes such as alcohol metabolism were inhibited in the high *GFRA3* expression group, when compared with the low *GFRA3* expression group (**Figures S5C, D**).

In LIHC with high *GRIN2D* expression, 1,971 genes were upregulated and 302 genes were downregulated (FC>2, *P*<0.05) (**Figures S6A, B**). On the other hand, the high *GRIN2D* expression group had an upregulation of pathways like proteoglycans in cancer, the PI3K-Akt signaling pathway, cell adhesion molecules, and activation of processes like the positive regulation of cell activation, regulation of leukocyte proliferation, downregulation of bile secretion, cholesterol metabolism, and suppression of processes like the alcohol metabolic process and lipid homeostasis (**Figures S6C, D**).

These findings suggest that *GHRNE* and *GFRA2* expression in LIHC might be beneficial in maintaining the liver physiological function and suppressing tumor growth and metastasis; the effect of *GFRA3* and *GRIN2D* was antagonistic to *GHRNE* and *GFRA2*.

### Correlation Between CHRNE/GFRA2/GFRA3/GRIN2D Expression and Immune Infiltration, Immune Response, and Stemness

**Figure 7:**
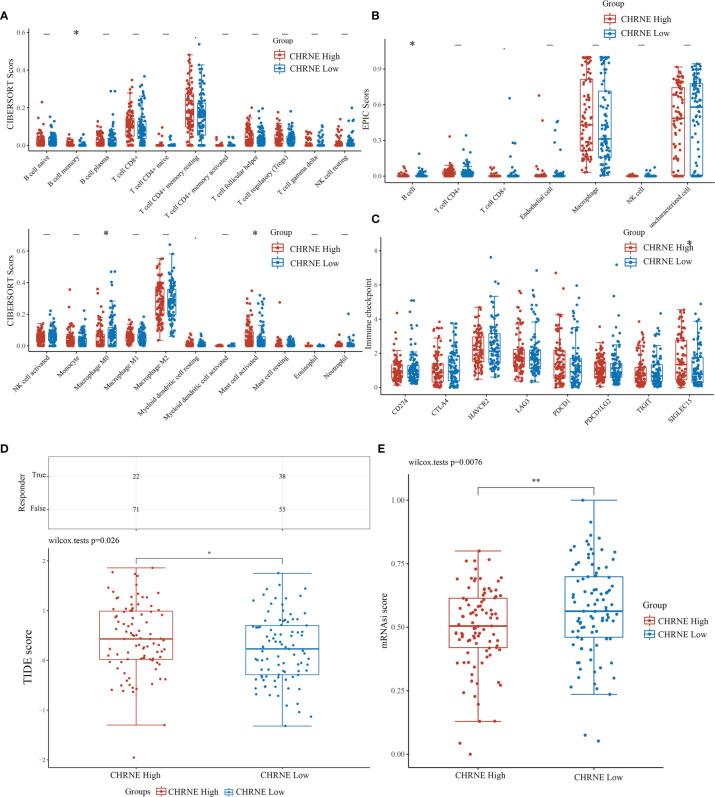
Comparisons of immune status and stemness between CHRNE high expression group and low expression group. **(A, B)** Comparison of CHRNE high expression group and CHRNE low expression group in immune infiltration obtained with CIBERSORT and EPIC algorithm; The horizontal axis represents different immune cells, the vertical axis represents the immune scores (*P < 0.05, **P < 0.01). **(C)** Comparison immune checkpoint genes expression in CHRNE high expression group and CHRNE low expression group; The horizontal axis represents different immune checkpoint genes, the vertical axis represents the expression level (*P < 0.05). **(D)** Statistical table of immune response and the distribution of immune response scores of the different groups in predict results. (*P < 0.05) **(E)** Comparison of CHRNE high expression group and CHRNE low expression group in stemness was exhibited by mRNAsi score with OCLR algorithm. (**P < 0.01).

The R software package, immunedeconv, was used to obtain the immune infiltration data of high/low-expression *CHRNE*, *GFRA2*, *GFRA3*, and *GRIN2D* groups of *LIHC*. CIBERSORT and EPIC algorithms were used herein. The CIBERSORT algorithm showed that in C1, unlike C2, high *CHRNE* expression was positively correlated with memory B cell (*P*<0.05) and mast cell (activated/resting) infiltration (*P*<0.05), while being negatively correlated with macrophage M0 (*P*<0.05) (**Figure 7A**). The EPIC algorithm showed that in C1, compared with C2, high *CHRNE* expression was positively correlated with B cell (*P*<0.05) (**Figure 7B**). In terms of high *GFRA2* expression, the CIBERSORT algorithm showed that in C1, compared with C2, it was positively correlated with the CD4+ memory resting T cell (*P*<0.001), while it was negatively correlated with monocytes (*P*<0.05), macrophage M0 (*P*<0.001), eosinophils (*P*<0.05), and neutrophils (*P*<0.01) (**Figure S7A**). The EPIC algorithm showed that high *GFRA2* expression was positively correlated with the CD4+ T cell (*P*<0.001) (**Figure S7B**). The CIBERSORT algorithm showed that in C1, compared with C2, high *GFRA3* expression was positively correlated with the memory resting B cell (*P*<0.01) and T cell follicular helper (*P*<0.01), while it was negatively correlated with monocytes (*P*<0.001) (**Figure S8A**). The EPIC algorithm showed that low *GFRA3* expression was positively correlated with macrophages (*P*<0.001) (**Figure S8B**). The CIBERSORT algorithm showed that in C1, compared with C2, high *GRIN2D* expression was positively correlated with Tregs (*P*<0.05) and macrophage M0 (*P*<0.001), while it was negatively correlated with naïve B cells (*P*<0.01), resting natural killer (NK) cells (*P*<0.001), monocytes (*P*<0.001), and activated mast cells (*P*<0.01) (**Figure S9A**). The EPIC algorithm also showed that high *GRIN2D* expression was positively correlated with the CD4+ T cell (*P*<0.001), but negatively correlated with macrophage (*P*<0.001) (**Figure S9B**).

In addition, we analyzed the correlations between ICGs and the expression of *CHRNE*, *GFRA2*, *GFRA3*, and *GRIN2D*. When compared with C2, *CHRNE* expression in C1 was positively correlated with *SIGLEC15* (*P*<0.05) (**Figure 7C**); *GFRA2* and *GRIN2D* expression was positively correlated with 7 of 8 IGCs including *CD274*, *CTLA4*, *HAVCR2*, *LAG3*, *PDCD1*, *PDCD1LC2*, and *TIGIT* with significant difference (*P*<0.001) (**Figures S7C**, **S9C**); *GFRA3* was positively correlated with *CTLA4* (*P*<0.001), *HAVCR2* (*P*<0.01), *LAG3* (*P*<0.001), *PDCD1* (*P*<0.001), and *TIGIT* (*P*<0.01) (**Figure S8C**). The TIDE algorithm showed that high expression of *CHRNE*, *GFRA3*, and *GRIN2D* correlated with a poor immune response (**Figures 7D**, **S7D**–**S9D**). According to the Spearman correlation analysis of the OCLR score, the *CHRNE* (**Figure 7E**), *GFRA2* (**Figure S7E**), and *GRIN2D* (**Figure S9E**) high-expression groups show a lower stemness score than the low-expression groups, whereas *GFRA3* (**Figure S8E**) has an opposite result.

### Gene Landscape of CHRNE/GFRA2/GFRA3/GRIN2D

**Figure 8:**
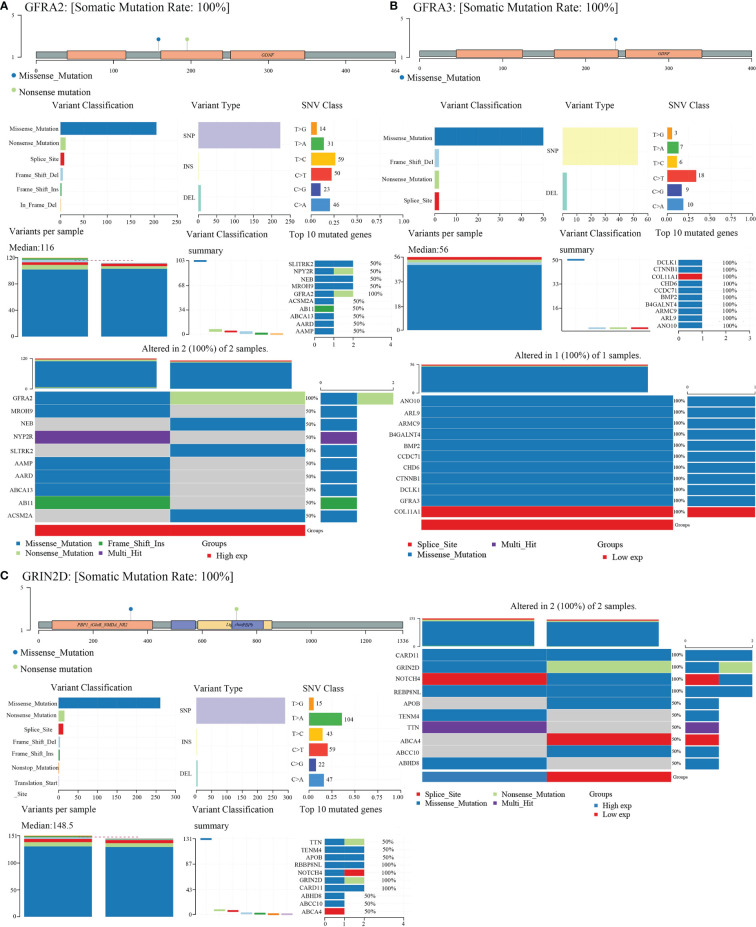
Gene mutation landscape of *GFRA2*, *GFRA3*, and *GRIN2D*. **(A)** Gene mutation landscape of *GFRA2*. A lollipop plot, an oncoplot, and cohort summary plot are shown to display the distribution of gene mutation. **(B)** Gene mutation landscape of *GFRA3*. **(C)** Gene mutation landscape of *GRIN2D*.

We obtained mutational, transcriptomic, and clinical data of LIHC patients from the TCGA database and performed visualization analysis with R software package maftools (17) and found no significant mutations for *CHRNE*, *GFRA2*, *GFRA3*, and *GRIN2D* in LIHC (**Figure 8**).

## Discussion

Neural infiltration has been viewed as a crucial aspect of the tumor microenvironment, which had also been termed as the innervated niche (7, 18). Together with the hypoxic niche, immune microenvironment, metabolic microenvironment, acidic niche, and mechanical microenvironment, neural infiltration regulates a series of biological processes in cancer cells and non-malignant cells in the microenvironment, which then influence cancer growth and metastasis. However, the complex neuroanatomy and intricate nature of the nervous system largely hinder further studies on nerve–cancer crosstalk.

Precision medicine is the future of cancer diagnosis and treatment, and the establishment of cancer subtypes based on gene expression has been proven to guide clinical practice. A typical example is the classification of breast cancer based on *Her2* and estrogen receptor expression. In this study, we classified LIHC into two subtypes, C1 and C2, based on NRGs. C1 and C2 had statistical differences in prognosis as well as a significant difference in unregulated/downregulated signaling pathways and biological processes. Immune infiltration and ICG analysis showed a notable discrepancy between the immune microenvironments of C1 and C2. Furthermore, the TIDE algorithm confirmed the immune response differences between the two subtypes. These findings confirm the close connection between neural infiltration and liver cancer and indicate a reclassification of liver cancer based on NRGs as a promising avenue for translational into a clinical setting.

We also screened out four NRGs (*CHRNE*, *GFRA2*, *GFRA3*, and *GRIN2D*) in LIHC using a prognostic model. *CHRNE* is the acetylcholine receptor subunit epsilon (ϵ-AChR) engaged in maintaining the normal function of neuromuscular junction (19–21). Mutations in *CHRNE* were reported to be associated with the myasthenic syndrome; however, it was never associated with cancer. Our analysis showed that *CHRNE* was related to cancer-associated signaling pathways, including PI3K-Akt signaling pathways and liver metabolic pathways. Clinical data showed that *CHRNE* expression was correlated with the T category and LIHC prognosis. We proposed that *CHRNE* might build a bridge between nerve cells and cells in the tumor microenvironment and influence cancer progression. *GFRA2* and *GFRA3* were glial cell line-derived neurotrophic factor (GDNF) receptors (22–24), and previous studies have suggested their participation in cancer. *GFRA2* interacts with *PTEN*, activates the PI3K/AKT pathway, and promotes neuroblastoma cell proliferation (22). On the other hand, *GFRA3* promoted the proliferation and invasion of pancreatic ductal adenocarcinoma cells (25), and its expression was negatively correlated with urothelial carcinoma prognosis (26). Additionally, genome-wide DNA methylation profiling showed that *GFRA3* promoter methylation was negatively correlated with gastric cancer prognosis (27). However, in our analysis, *GFRA2* exhibited an anti-tumor effect, while *GFRA3* exerted a pro-tumor effect. *GRIN2D* encoded N-methyl-D-aspartate receptor (NMDAR) subunit ϵ-4, which interacted with *NMDA* and was involved in developmental and epileptic encephalopathy (28, 29). It was regarded as the biomarker for colorectal cancer angiogenesis (30). Our study suggested that *GRIN2D* is clinically significant and holds a biological value in liver cancer. Importantly, *GRIN2D* may serve as a potential biomarker to assess therapeutic responses to ICBs owing to a strong correlation with IGCs (*e.g*., *CD274*, *CTLA4*, *LAG3*) and immune cell (*e.g.*, B cell, T cell CD4+, NK cell) infiltration. We concluded that *GRIN2D* exerted an immunomodulatory role on the tumor microenvironment *via NMDA* targeting and consequently influence cancer proliferation and metastasis. Therefore, the blockade of *GRIN2D* may help sensitize patients’ immune response.

To summarize, our study attempted to uncover the role of NRGs in LIHC and highlight that their importance in cancer progression demonstrated that NRGs play an important role liver cancer growth and migration, immune infiltration, immune response, and the upregulation or downregulation of clinically significant pathways. Specific NRGs, *CHRNE*, *GFRA2*, *GFRA3*, and *GRIN2D*, could serve as potential biomarkers for LIHC prognosis. However, basic experiments and clinical trials are both required to verify the inferences drawn from bioinformatics analyses.

## Data Availability Statement

Publicly available datasets were analyzed in this study. This data can be found here: https://www.cancer.gov/about-nci/organization/ccg/research/structural-genomics/tcga.

## Author Contributions

Y-GZ and X-RZ did the analysis. Y-GZ and M-ZJ wrote the paper. X-RZ did the data sorting and charting. W-LJ conceived the paper. All authors contributed to the article and approved the submitted version.

## Funding

This study was supported by the First Hospital of Lanzhou University for the introduction of high-level talents to start research funding.

## Conflict of Interest

The authors declare that the research was conducted in the absence of any commercial or financial relationships that could be construed as a potential conflict of interest.

## Publisher’s Note

All claims expressed in this article are solely those of the authors and do not necessarily represent those of their affiliated organizations, or those of the publisher, the editors and the reviewers. Any product that may be evaluated in this article, or claim that may be made by its manufacturer, is not guaranteed or endorsed by the publisher.

## Supplementary Material

Supplementary Figure 1 Comparisons of 38 neural-related gene expression between C1 and C2.

Supplementary Figure 2 The construction of a prognostic model based on 4 differentially expressed neural-related genes.

Supplementary Figure 3 Comparisons of clinical characteristics between high and low *CHRNE/GFRA2/GFRA3/GRIN2D* groups. Comparisons of T category, N category, M category, TNM staging, and pathological grading between **(A)** *CHRNE*-high and -low group; **(B)** *GFRA2*-high and -low group; **(C)** *GFRA3*-high and -low group; **(D)** *GRIN2D*-high and -low group.

Supplementary Figure 4 Differential expression and enrichment analysis of high and low *GFRA2* expression groups.

Supplementary Figure 5 Differential expression and enrichment analysis of high and low *GFRA3* expression groups.

Supplementary Figure 6 Differential expression and enrichment analysis of high and low *GRIN2D* expression groups.

Supplementary Figure 7 Comparisons of immune status and stemness between high and low *GFRA2* expression groups.

Supplementary Figure 8 Comparisons of immune status and stemness between high and low *GFRA3* expression groups.

Supplementary Figure 9 Comparisons of immune status and stemness between high and low *GRIN2D* expression groups.
