# Supplementary figures and images for: Reclassification of Hepatocellular Cancer With Neural-Related Genes
Source: Front Oncol. 2022 May 13;12:877657. doi: 10.3389/fonc.2022.877657 (PMC9136183; doi:10.3389/fonc.2022.877657)

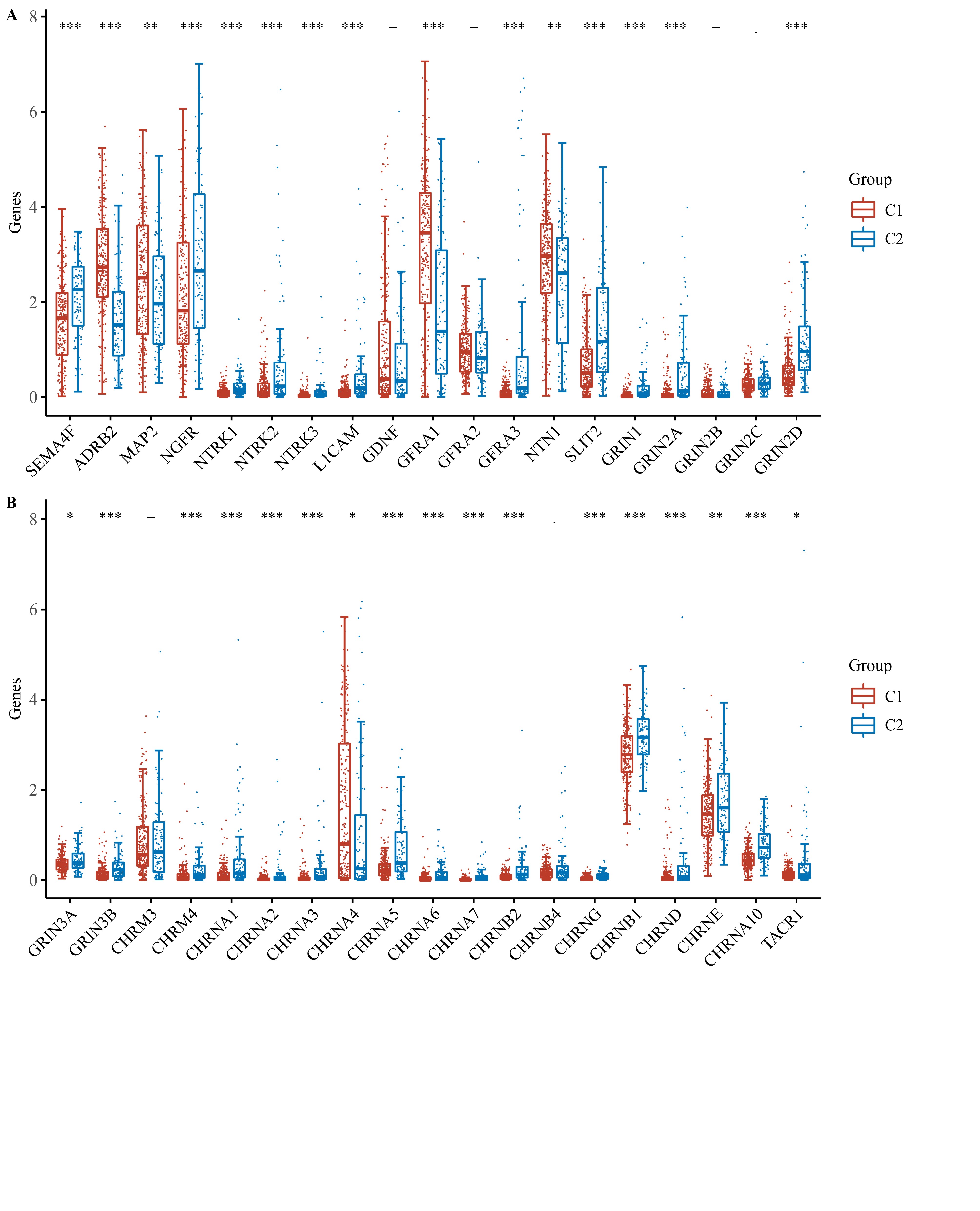

Supplement: Supplementary Figure 1 — Comparisons of 38 neural-related gene expression between C1 and C2. [file Image_1.jpeg]

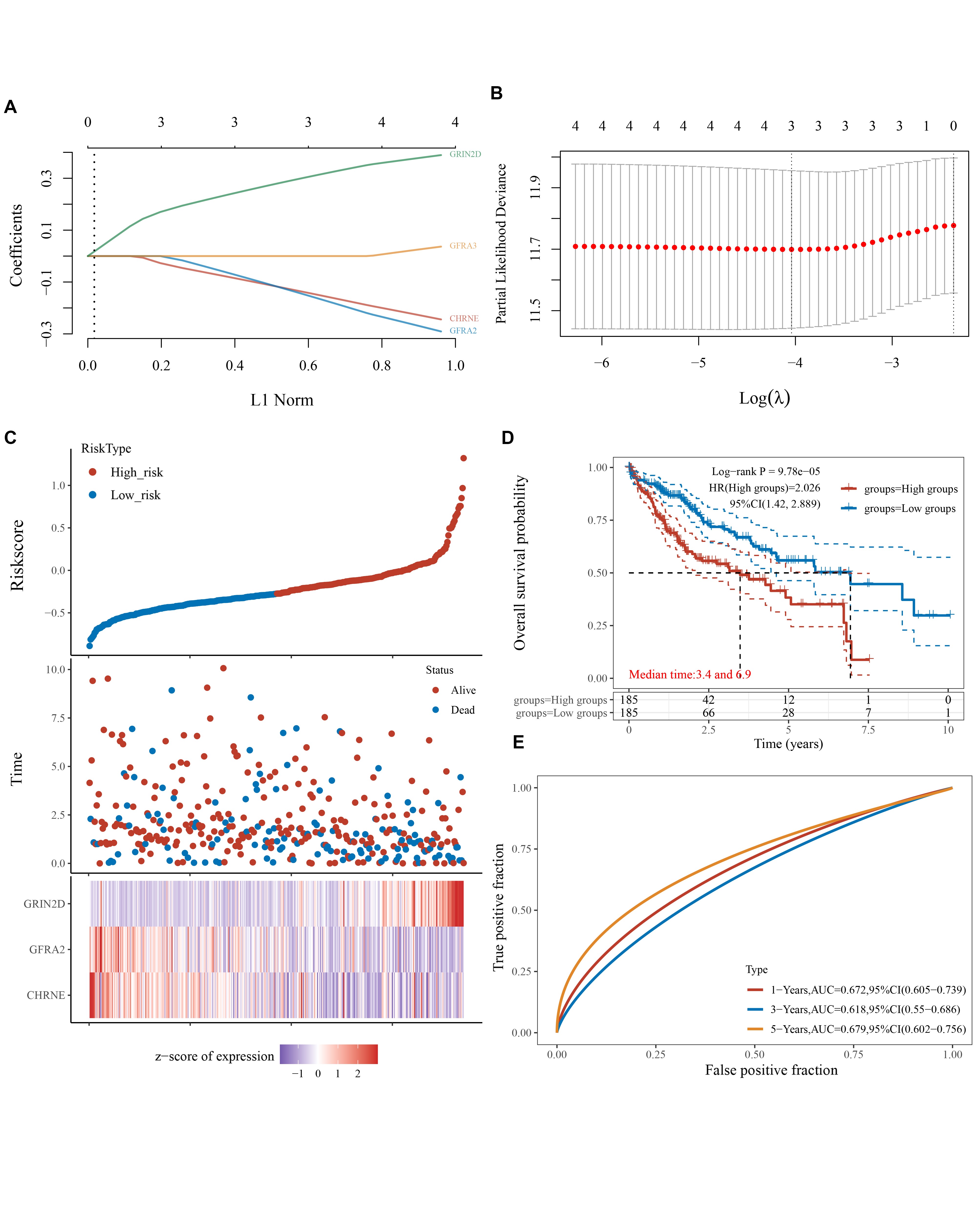

Supplement: Supplementary Figure 2 — The construction of a prognostic model based on 4 differentially expressed neural-related genes. [file Image_2.jpeg]

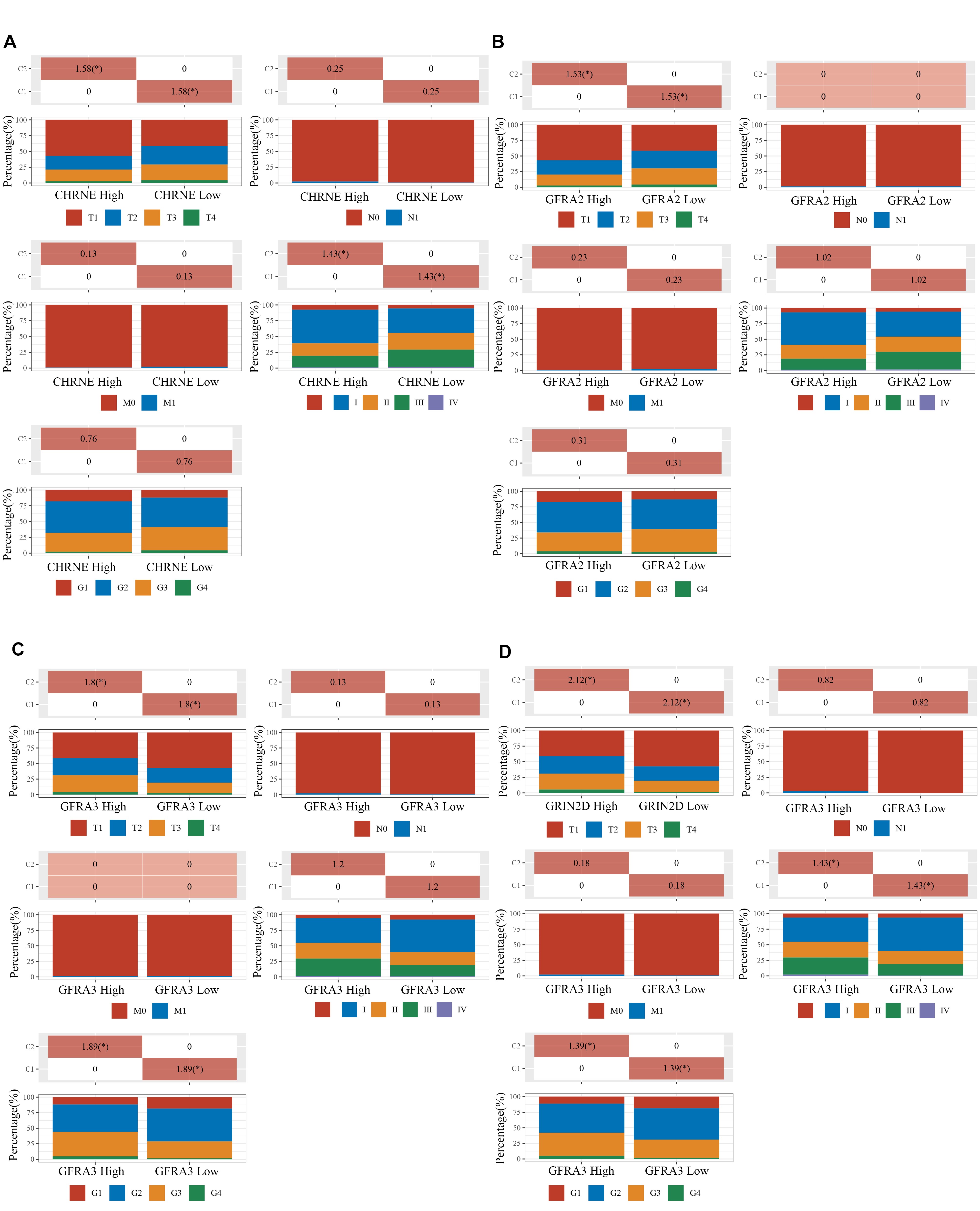

Supplement: Supplementary Figure 3 — Comparisons of clinical characteristics between high and low CHRNE/GFRA2/GFRA3/GRIN2D groups. Comparisons of T category, N category, M category, TNM staging, and pathological grading between (A) CHRNE-high and -low group; (B) GFRA2-high and -low group; (C) GFRA3-high and -low group; (D) GRIN2D-high and -low group. [file Image_3.jpeg]

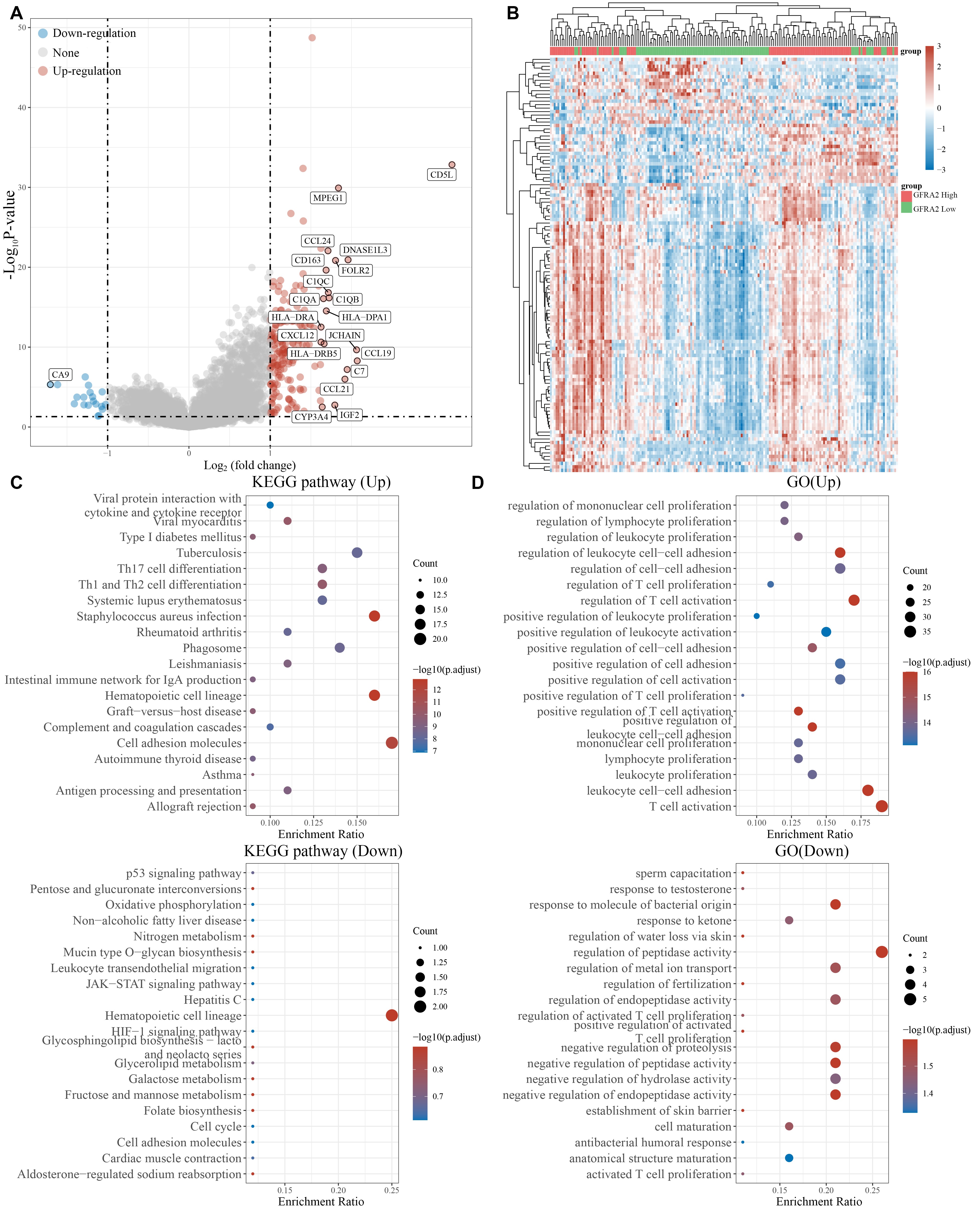

Supplement: Supplementary Figure 4 — Differential expression and enrichment analysis of high and low GFRA2 expression groups. [file Image_4.jpeg]

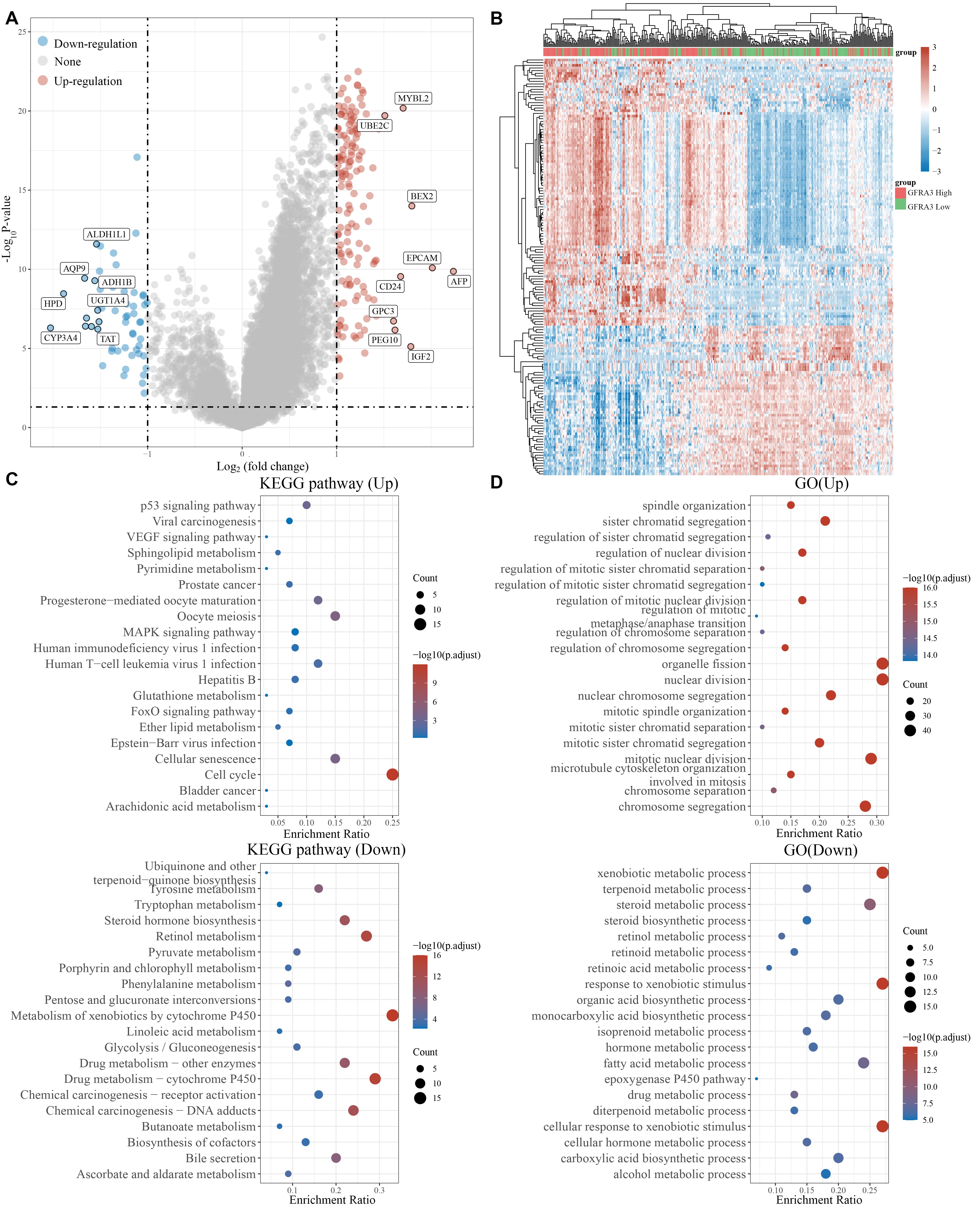

Supplement: Supplementary Figure 5 — Differential expression and enrichment analysis of high and low GFRA3 expression groups. [file Image_5.jpeg]

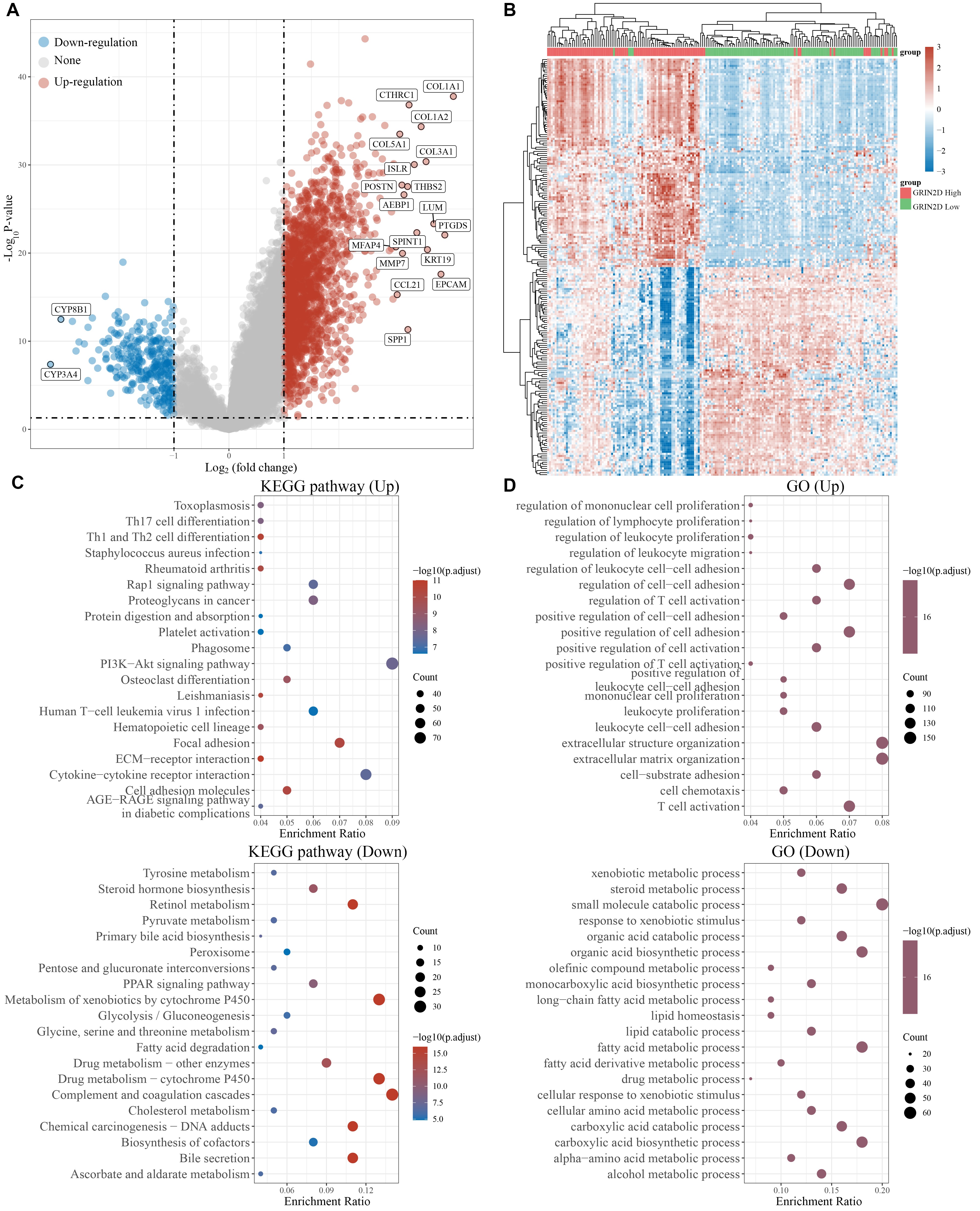

Supplement: Supplementary Figure 6 — Differential expression and enrichment analysis of high and low GRIN2D expression groups. [file Image_6.jpeg]

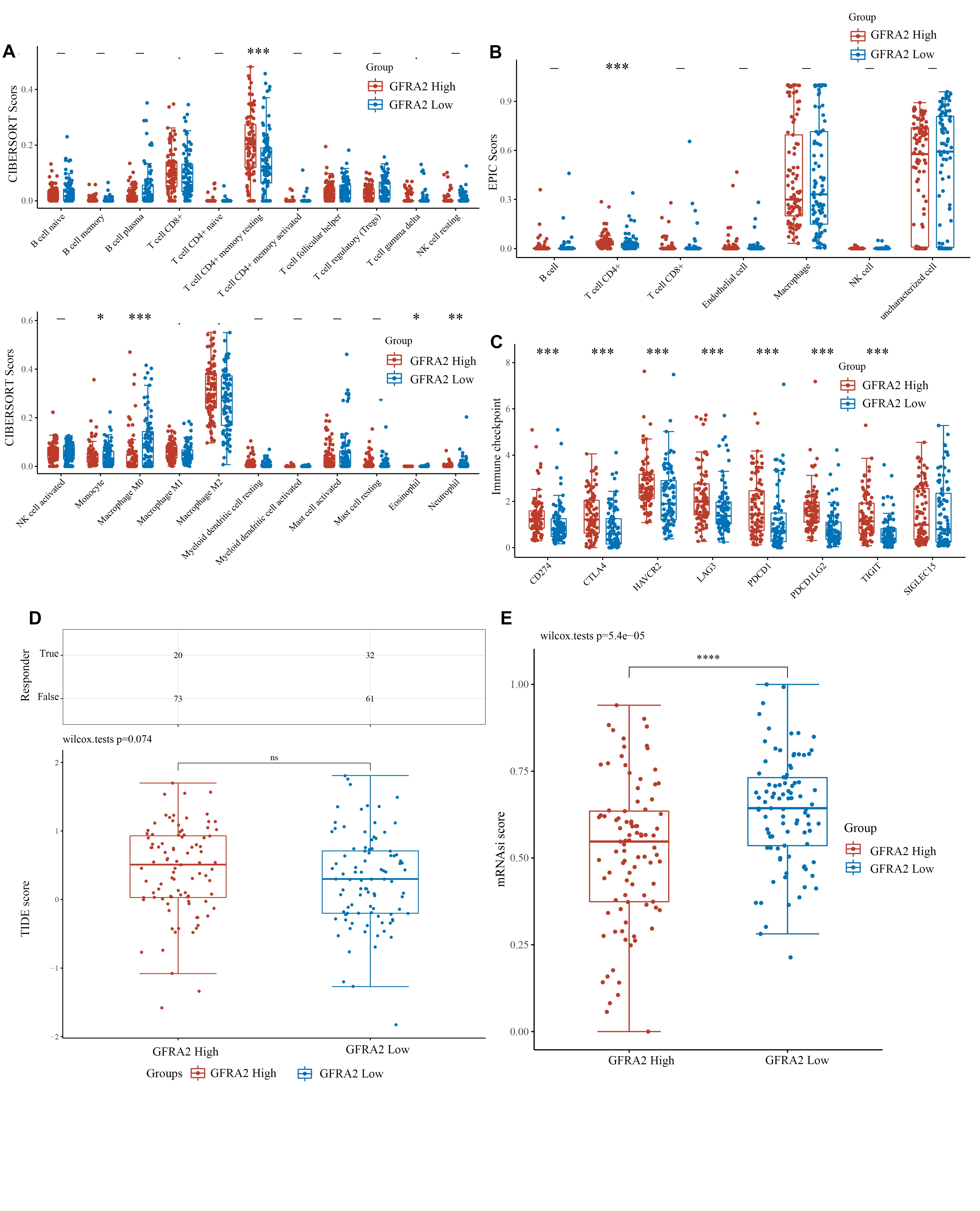

Supplement: Supplementary Figure 7 — Comparisons of immune status and stemness between high and low GFRA2 expression groups. [file Image_7.jpeg]

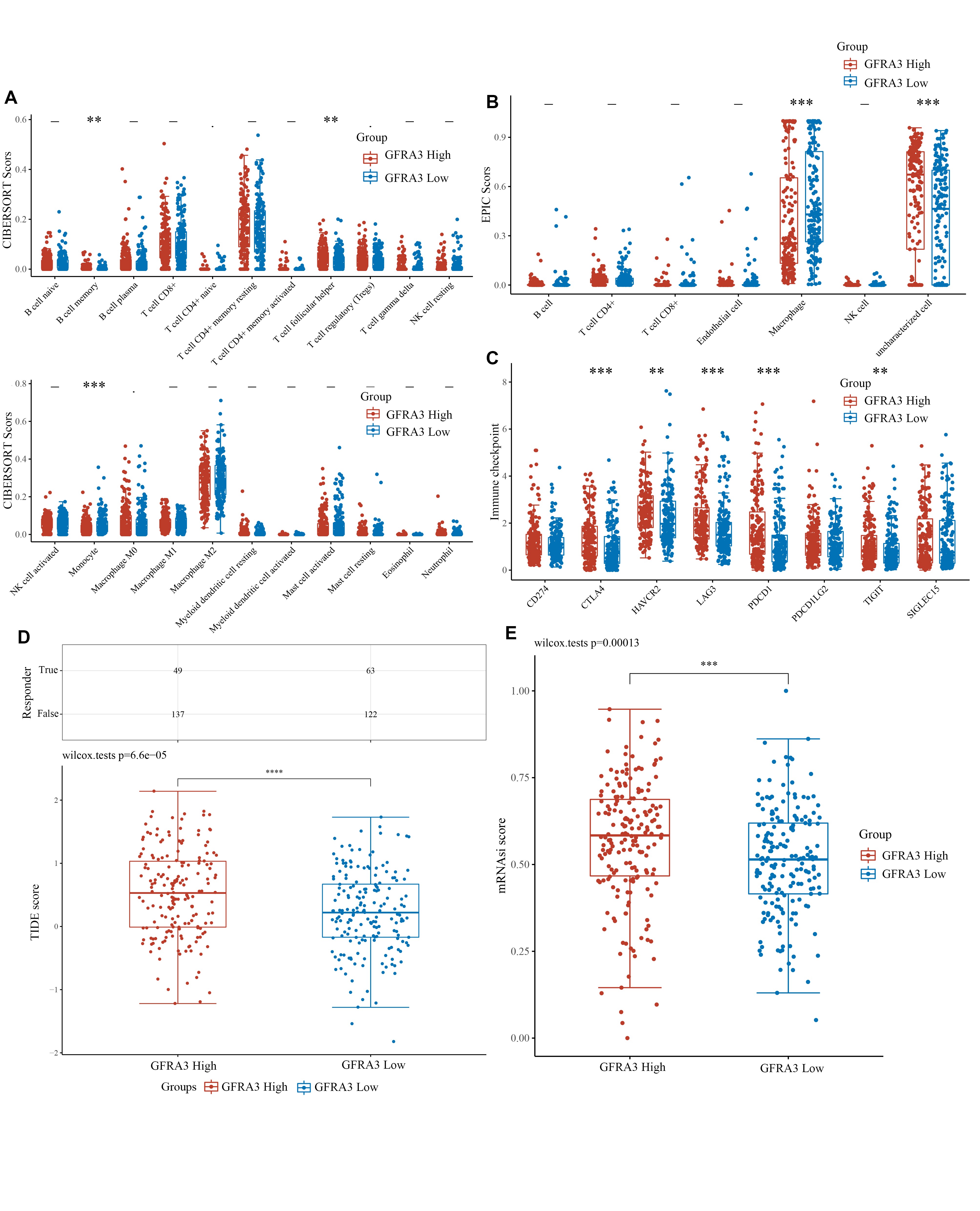

Supplement: Supplementary Figure 8 — Comparisons of immune status and stemness between high and low GFRA3 expression groups. [file Image_8.jpeg]

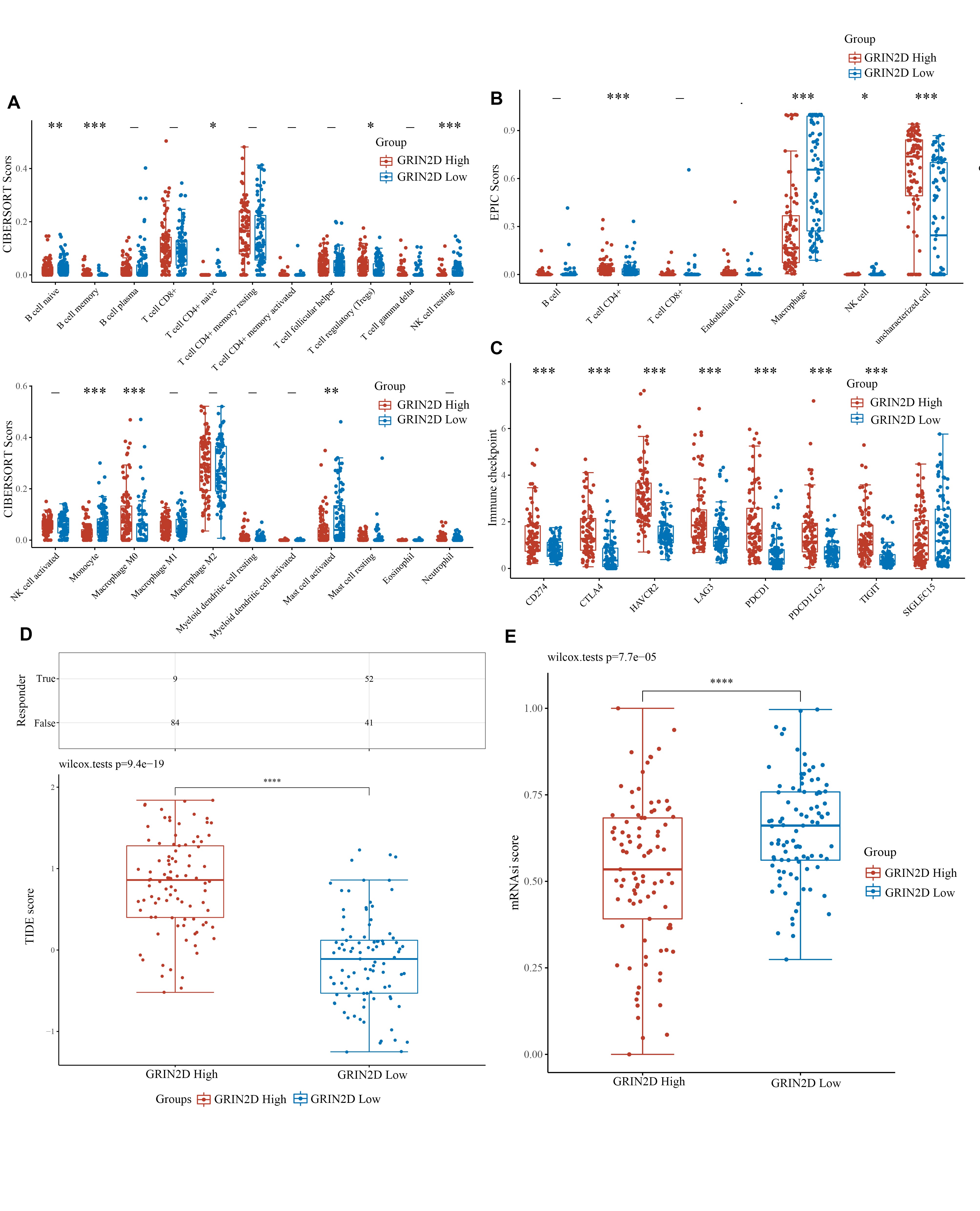

Supplement: Supplementary Figure 9 — Comparisons of immune status and stemness between high and low GRIN2D expression groups. [file Image_9.jpeg]
